# 

**Published:** 1883-12

**Authors:** 


### TO OUR SUBSCRIBERS.

With this number will expire a large number of subscriptions to the INDEPENDENT PRACTITIONER. We hope that there will be very few of them who will not desire the journal for the coming year. If we do not make it worth ten times the price of subscription, it will be because some unforeseen catastrophe awaits us. Its proprietors have in store some announcements, which they hope to make in the January number, that will be a guarantee of its future excellence. We might publish, from men of the highest standing in the profession, letters of commendation and expressions of their appreciation of the present value of the journal, but we prefer to let it speak for itself.

We hope that such as desire it for the coming year will send in their subscriptions at once, and thus secure its prompt reception. If there be any who wish its discontinuance, they should notify us promptly. Subscription blanks will be enclosed to those whose term expires with the present number. Send all business communications to the New York office.

689

### INDEX

TO THE

### INDEPENDENT PRACTITIONER.

### VOLUME IV.

### ORIGINAL COMMUNICATIONS.

Abscesses of the Ano-Perineal Region. Surg. Gen. Philip S. Wales..... 249
Action of Arsenious Acids on Dental Tissues. Dr. C. F. W. Bodecker..... 468
Æsthetic Dentistry. Dr. E. H. Bunting............................ 460
American Dental Association—Again. K.—H. ........................ 645
Anatomy of the Human Tooth, Minute. Prof. Frank Abbott...... .... 535
Application of Nitrous Oxide and Air under pressure. Dr. E. P. Howland. 455
Antiseptic Treatment of Diseases of the Dental Pulp. Dr. E. Rosenthal. 585

Caries of Human Teeth. Prof. Frank Abbott........................ 198
Caries of the Human Teeth. Dr. W. D. Miller......................... 629
Case in Practice, A. Dr. W. P. Richards............................ 650
Cause of Caries—Bacteria or Acids? Dr. F. Y. Clark..... ........... 132
Causes of Failure of Gold as a Filling Material. Dr. A. A. Blount...... 588
Copyright Law, The proposed. "Medicus Americanus."................ 318
Curse of Modern Civilization, The. Dr. G. Carleton Brown............ 526

Density of Enamel, On the. Dr. J. Edw. Line........................ 349
Dental Caries. Dr. W. D. Miller................................260, 301
Dental Caries, The Germ Theory of. Dr. C. T. Stockwell ............ 73
Dental Periostitis, The Grindstone Cure. Dr. J. Requa................ 529
Difficult Dentition and the use of the Gum Lancet. Dr. J. Morgan Howe 573
Disease. Dr. W. H. Atkinson ......................................... 311
Diseases of the Dental Pulp. Dr. C. F. W. Bodecker.................. 369

Electrical Phenomena in the Mouth. Dr. W. D. Miller ................ 86
Engines and Hand-Pieces, Something about. Dr. N. W. Kingsley ...... 582
Extraction of Deciduous Teeth. Dr. N. W. Kingsley................... 397

Filling Teeth and the Philosophy Thereof. Dr. J. Hayhurst............ 465

Garretson's Operation for removal of the Coccyx. Dr. S. Parker Cottrell.. 8
Gutta-Percha Stopping. Dr. C. E. Francis........ .................... 649

Keeping the Teeth Clean. Dr. C. E. Francis........................... 204

Legal and Moral Responsibility of Dentists. Dr. J. Allen Osmun.. ..... 404
Lithotritry at a Single Sitting. Prof. F. N. Otis....................... 1

Medical Clinic. Prof. Alfred L. Loomis........................129, 193
Medical Clinic. Prof. Jos. T. Whittaker........................ 65
Notes on Operative Dentistry—its Advancement. Dr. G. H. Perine 591, 652
Parotid Gland, Removal of. Dr. Claude Browning.................... 10
Per-Oxide of Hydrogen ($H^2$ $O^2$). Dr. A. W. Harlan.................. 138
Practical Hints for Dentists. Dr. J. A. Robinson...................... 139
Purulent Inflammation of the Middle Ear. Dr. Edward S. Peck......... 401
Rachitic Deformities, Some of the More Common. Prof. V. B. Gibney.. 4
Teeth of Pre-Historic American Races. Dr. W. C. Barrett.............. 513
Theory vs. Facts. Dr. A. M. Ross.................................. 412
Trephining for Depressed Fractures in Vertex of Skull. Dr. Parker Syms.................................................. 72
Use of the Screw in Regulating Teeth, The. Dr. Wm. H. Trueman..... 521
Wounds of the Eye-Ball. Dr. Peter A. Callan........................ 79

ORIGINAL TRANSLATIONS AND ABSTRACTS.

Adulterated Foil.................................................. 341
Alveolar Dilator, An.............................................. 342
Amalgams, Composition of Different................................ 541
Cerebral Tumor.................................................... 90
Chronic Complaint, A.............................................. 374
Chronic Diphtheria of the Pharynx................................. 81
Definitions....................................................... 471
Dental Prosthesis................................................. 371
Dental Society of the State of New York........................... 289
Dentist's Exhibits, German Prize for.............................. 542
Dentist's Instruments............................................. 415
Discussion of the Treatment of Deciduous Teeth.................... 146
Drill, Hard Tempered.............................................. 542
Epilepsy, Cure of, by Ligature of Vertebral Arteries.............. 91
Expensive Treatment............................................... 294
Extraction with Gas............................................... 343
Foreign Bogus Diploma Mills....................................... 340
Fungi of Tooth Caries............................................. 210
German Dentistry.................................................. 342
Gold Foil in Germany.............................................. 293
Good for Mexico................................................... 470
Hemorrhage after Tooth Extraction................................. 292
Metal Plates, A new method for the manufacture of................. 415
Muse in Medicine, The............................................. 91

Plastic Filling Materials, Experiments with Various.................... 417
Preservative Fluid........ :........................................ 542
Product of the Hen.................................................. 471
Propagation of Bacteria.... ........................................ 340
Pyonephrosis, An Operation for.................................142, 206
Rust, To Remove..................................................... 415
Spasm of the Voice, A rare Case of.................................. 13
Snake Bite. Rapid Cure by Carbolic Acid............................. 13
Toothache, Kidney Trouble the Cause of.............................. 541
Tumor of the Left Supra-Renal Capsule...... ........................ 12
What a Record....................................................... 342

CORRESPONDENCE.

British Dental Association. Dr. E. A. Bogue........................ 532
Dental Patents and Remedies. Dr. Ambler Tees........................ 673
Letter from Geneva, Switzerland. Dr. A. A. Blount................... 616
Letter from Rome, Italy. Dr. J. G. Van Marter....................... 673
The Independent Practitioner. Dr. J. W. Clowes...................... 617
Teeth of Early Egyptians. Dr. E. T. Darby........................... 671
Webb's Notes on Operative Dentistry. S. S. White Dental Mfg. Co..... 381

REPORTS OF SOCIETY MEETINGS.

American Dental Association......................................... 489
American Dental Society of Europe........................... 495, 602, 656
Central Dental Association of Northern New Jersey..............354, 418
Connecticut Valley Dental Society................................... 667
Dental Society of the State of New York............................. 289
New England Dental Society.......................................... 612
New Jersey State Dental Society................................472, 542
Ohio State Dental Society........................................... 661
Pennsylvania State Dental Society..............................552, 594

EDITORIAL.

Adding Strength to Strength......................................... 683
American Dental Association......................................... 429
American Dental Association, The.................................... 497
American Medical Association, The................................... 378
Apology, An......................................................... 284
Attention........................................................... 562

**B**altimore Infectious Diseases Ordinance........ 43
Blessed Doctrine of Rest, The........ 375

**C**arbolic Acid, Another Use for........ 430
Carbolic Acid........ 45
Caries of Human Teeth........ 621
Change, A........ 380
Combined Filling........ 683
Convenience, A........ 175
Correction........ 501
Credit to Whom Credit is Due........ 682

**D**angerous Remedy, A........ 225
Dental Calendar........ 429
Dental Education........ 675
Dental Pathology........ 222
Diseases of Pre-Historic Man........ 559
Dr. Miller's Latest Investigation........ 679

**E**ditorial Amenities........ 49
Etiological Status, The Present........ 325
Etiology of Dental Caries........47, 175
Errata........ 431
Erroneous........ 562
Exchanges, Our........ 285
Explanatory........ 328

**F**acial Neuralgia........ 423

**G**old Alloy........ 109
Good Suggestion, A........ 328

**H**ardening Plaster Casts........ 285
Hear Both Sides........ 682
Hospital Saturday and Sunday........ 42

**I**ntroductory........ 40
INDEPENDENT PRACTITIONER, The........ 224
Is Dental Caries a Modern Disease........ 680

**K**och's Investigations, One Result of........ 222

**L**aboratory Lathes........ 620
Leigh H. Hunt, Dr........ 284
Liberal Offer, A........ 333
Lips of Patients, The........ 619
Look Ahead, A........ 283
Look Ahead, Another........ 329

**M**achinery .......... 621
Medical Literature and Medical Journals .......... 101
Microscopic Organisms and Antisepsis .......... 103

**N**ew Anæsthetic, A .......... 222
New Department .......... 329
New Medical Code, The .......... 282

**O**perations for Removal of the Kidney .......... 174
Our Journal .......... 621

**P**ain Obtunders .......... 223
Pasteur's Experiments Concerning Contagious Diseases .......... 173
Pearl in the Oyster, The .......... 501
Platinum and Gold Blocks .......... 176
Prone Position for the Administration of Anæsthetics, The .......... 618
Publisher's Notice .......... 49, 225

**Q**uestions of Interest to the Dental Specialist .......... 49

**R**elaxation for Dentists .......... 104
Resignation, Dr. Hunt's .......... 221
Respect Our Identity .......... 683
Retrospective and Prospective .......... 677

**S**alutational .......... 40
Save your Eyes .......... 48
Singular Cause of Death .......... 48
Sponges for Dentists' Use .......... 110
Springfield Meeting, The .......... 332
Subscribers, To .......... 502

**T**oothache .......... 379
To Our Subscribers .......... 681
To Young Practitioners .......... 277
Typhoid Epidemic in Paris, The Recent .......... 44

**U**niversity of Buffalo .......... 428
Use of the Gum Lancet, The .......... 170

**V**isiting Lists .......... 682

### NEW APPLIANCES AND MATERIALS.

**A**bbott's Scissors .......... 622
Appointment Book, Pearson's .......... 623

**B**ur and Drill Gauge, New .......... 564

**D**ental Plaster .......... 563
Disc Carrier, New .......... 563

Enamel Chisels.......... 502
Impression Cups.......... 502
Kearsing's Foil.......... 623
Laboratory Furnace, New.......... 432
Liquid Gas Apparatus and Dental Cabinet, New.......... 431
Listerine.......... 503
Oxford Tooth Co., The.......... 562
Oxy-Phosphate Cement.......... 623
Pneumatic Plugger.......... 431
Robinson's Fibrous and Textile Metallic Filling.......... 381
Sandpaper Disc Cutter.......... 380

BIBLIOGRAPHICAL.

Bibliographical..........54, 114, 180, 229, 288, 337, 381, 433, 503

OBITUARY.

Beard, George M., A. M., M. D.......... 118
Beard, Mrs. Elizabeth A.......... 186
Buckingham, Thomas Lea, M. D., D. D. S.......... 564
Davaine, M.......... 58
Goddard, W. H., D. D. S.......... 233
McCall, H. S., M. D., M. D. S.......... 58
Ranney, Lafayette, M. D.......... 186
Van Buren, W. H., M. D., L. L. D.......... 231
Watson, Sir Thomas.......... 57
Webb, Marshall H., D. D. S..........119, 185

SELECTIONS AND EXTRACTS.

Addison's Disease.......... 188
Bacillus of Whooping-Cough, The.......... 238
Bacteria.......... 33
Cancer of the Face, Extirpation of.......... 210
Case of Tumor, A.......... 234
Celluloid.......... 571
Cincinnati.......... 572
Consumption; How it may be Prevented.......... 35
Development of Disease Germs.......... 92
Dr. Beard on Herbert Spencer.......... 235

Dropsy of the Antrum.......................................... 386
Dropsy of the Antrum; Punctured and Drained; Cure.................. 445

Effects of Division of the Vagi upon the Heart.......................... 167
Effect of Tobacco on Children, The .................................. 447
Extension of Vice, The................................................ 450
Extra Uterine Pregnancy, Thomas on.................................. 188

Facial Neuralgia.................................................. 442
Faith as an Element of Success in Medicine.............................. 37
Fangs of the Rattlesnake............................................ 237
Freckles.......................................................... 510

Gold vs. Amalgam.................................................. 213
Good Diagnosis.................................................... 450

Honest and True New York Doctors..................................... 236
Hot Water as a Beverage............................................. 324
Hyperidrosis...................................................... 572

Introductory Lecture to the Course of Therapeutics....................... 15

Koch's Bacillus and its Lessons........................................ 215

Life of Man, The.................................................. 510
Locomotor Ataxy, Lesions of the Teeth in............................... 122

Micro-Organism of Whooping-Cough, The.................................. 237

Nerve Instruments.................................................. 510
New Anæsthetic—Chloroform, Ether, and Absolute Alcohol................. 97
New Anatomy Act, The............................................... 219
New Idea for the Spread of the Knowledge of Medical Ethics, A........... 236
Nitrous Oxide in Labor. ............................................. 449
Notched Teeth...................................................... 511
Notes upon a Method of Root-Filling..................................... 569

Ozone as an Anæsthetic and Hypnotic.................................... 508

Perinephritic Abscess............................................... 165
Placenta Prævia.................................................... 123
Pleurisy in Children.................................................. 21
Pneumonia of Early Infancy, Pathology and Treatment of.................. 152
Polio-(Trepho)-Myelitis Anterior....................................... 187
Popliteal Aneurism, Radical Cure of.................................... 121
Pulpless Teeth, A Class of............................................. 28

Removal of a Maxillary Tumor with the Dental Engine..................... 448

Scarlatina and Diphtheria, Close Relation of............................ 160
Senses in New Born Infants........................................... 388

Severe Hemorrhage after Tooth Extraction.......................... 323
Statistics of Symphysiotomy.......................... 238
Swallowing False Teeth.......................... 322
Syphilis in the Ninth Century.......................... 389

Teeth in Diabetes, The.......................... 321
Test for Albumen, A Convenient and Delicate.......................... 122
Terms of Pupilage.......................... 450
Tooth at Birth, A.......................... 449
Treatment for Neuralgia, The Mechanical.......................... 123
Treatment of Ulcers.......................... 237
Trigeminus Nerve, Influence on the Organs of Hearing.......................... 100
Typhoid Fever, Antiseptic Treatment of.......................... 38
Typhoid Fever and Salicylic Acid.......................... 31

Use of Tooth Brushes, The.......................... 570

Vegetable Nature of Croup.......................... 270
Vomiting of Pregnancy.......................... 218

Why is Chloroform so Well Borne in Midwifery?.......................... 234

CURRENT NEWS AND OPINION..............51, 54, 111, 114, 176, 180, 225, 231, 286, 288, 333, 337, 383, 386, 436, 442, 504, 509, 566, 569, 624, 628, 683, 685, 686, 687, 648, 644

POPULAR SCIENCE..........................59, 64, 124, 128, 189, 192, 240, 248, 294, 299, 344, 348, 390, 396, 451, 454, 508, 512.

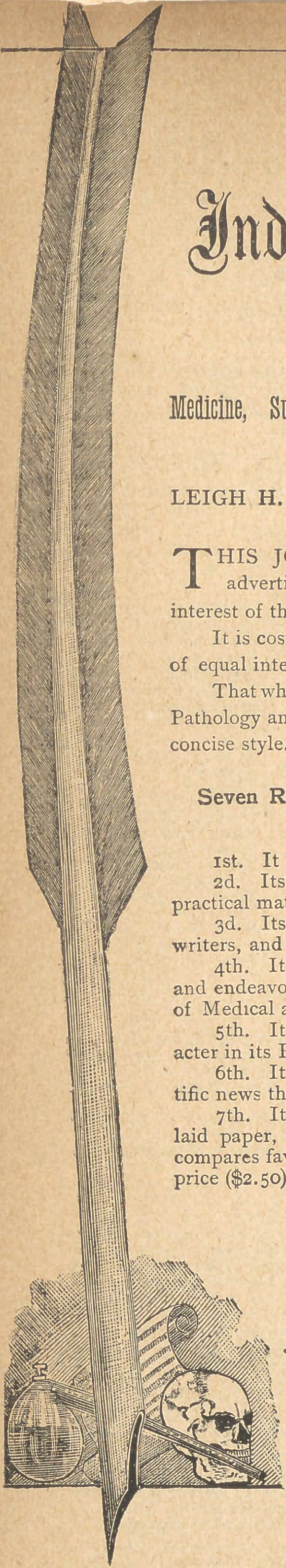

### PROSPECTUS

**For Vol. IV, 1883, of the**

### Independent Practitioner:

### A MONTHLY JOURNAL

DEVOTED TO

**Medicine, Surgery, Obstetrics, Dentistry, Pathology and Popular Science**

**EDITORS:**

LEIGH H. HUNT, B.Sc., M.D., W. C. BARRETT, D.D.S., M.D.

THIS JOURNAL is independent of colleges, cliques, isms, or any advertising firm; therefore, impartiality pervades its columns in the sole interest of the Profession.

It is cosmopolitan in its circulation and scientific resources; consequently of equal interest to the specialist and general practitioner everywhere.

That which is new and useful in Medicine, Surgery, Obstetrics, Dentistry, Pathology and Popular Science is given to its readers in an intelligent and concise style.

### Seven Reasons why every one interested in its Topics should subscribe now.

1st. It contains original papers from the best men in the Profession.

2d. Its translations, abstracts and selections contain more valuable and practical matter in an equal space than those of any other Journal published.

3d. Its Book Reviews are thorough criticisms made by competent writers, and in the interest of the Profession.

4th. It treats Dentistry as a specialty of general Medicine and Surgery, and endeavors to strengthen the relationship between the different branches of Medical and Surgical Science and Art.

5th. It furnishes original and selected matter of a high scientific character in its Popular Science Department.

6th. It gives notes and items of interest concerning medical and scientific news throughout the world, and the progress that has been made.

7th. It is printed with *new* Roman Long Primer type, on fine cream-laid paper, free from glaze, thereby giving relief to the eye of the reader; compares favorably with any Medical Journal published, and its subscription price ($2.50) is within the reach of all progressive practitioners.

*When ordered with one other Journal having a subscription price of $2.00 or more, the aggregate will be 50 cents less than the two subscribed for separately.*

*Send $2.50 without delay, in order to ensure a perfect set for 1883.*

*Specimen copies furnished upon application.*

**New York:**

**THE COLUMBIA PUBLISHING CO.,**

**42 Duane Street.**

### COMMENDATIONS.

The following extracts COMMENDATORY OF THE INDEPENDENT PRACTITIONER are taken from a large number of letters, from which we could fill pages with similar expressions:

PROF. ISAAC E. TAYLOR, M. D., President of Bellevue Medical College, New York, says: "I have been much pleased with the matter introduced, as well as the dress of the journal, and I wish you all success in the effort to advance the interests of the profession. I noticed a favorable criticism in the 'Medical Record' of last week."

PROF. ROBERTS BARTHOLOW, M. D., of the Jefferson Medical College, Philadelphia, says: "I shall be pleased to send you a paper when I get through the work which I am compelled to do."

PROF. J. M. DA COSTA, M. D., of the same college and city: "I wish it (the PRACTITIONER) a most marked success."

PROF. THOS. H. CHANDLER, Dean of the Harvard University, Dental Department: "If you keep up the standard, your magazine will fill a void in our professional literature."

PROF. JAS. E. GARRETSON, M. D., D. D. S., Dean of the Philadelphia Dental College: "Let me wish you success in an enterprise which looks as if calculated to be of great use to the dentist. I have read each number of the journal with much satisfaction and have to apologize for my tardiness in acknowledging your kindness in sending it. I welcome the PRACTITIONER particularly from the standpoint of its medico-dental character. The more dentists learn of general medicine and surgery the better are they found qualified for work as specialists."

COL. J. J. WOODWARD, Surgeon U. S. Army: "I wish for your enterprise a useful and honorable career and all success."

PROF. WM. A. HAMMOND, M. D., N. Y., and formerly Surgeon-General of the U. S. Army: "I am pleased with the INDEPENDENT PRACTITIONER and wish you success." Later: "I will ere long send you a paper which may prove of some interest to your readers."

PROF. ISAAC J. WETHERBEE, D. D. S., President of the Boston Dental College: "A careful examination of the contents of the INDEPENDENT PRACTITIONER leads me to believe that it will be a very useful publication to the dental profession, and I hope that you may meet with large success."

SAM D. RAMBO, D. D. S., of Rio, Brazil: "I find the INDEPENDENT PRACTITIONER very good. The best journal out."

Space will allow of the mention of a few more names only of those who have sent us words of encouragement or promised us original papers: PROF. AUSTIN FLINT, SR., M. D., PROF. FRANK H. HAMILTON, M. D., PROF. T. GAILLARD THOMAS, M. D., ALFRED L. LOOMIS, M. D., J. MARION SIMS, M. D., GEO. M. BEARD, and others in our city. PROF. WM. PEPPER, M. D., PROF. S. WEIR MITCHELL, M. D., RICHARD J. DUNGLISON, M. D., and PROF. CHAS. J. ESSIG, D. D. S., M. D., of Philadelphia.

### COMMENDATIONS.

The following extracts COMMENDATORY OF THE INDEPENDENT PRACTITIONER are taken from a large number of letters, from which we could fill pages with similar expressions:

PROF. ISAAC E. TAYLOR, M. D., President of Bellevue Medical College, New York, says: "I have been much pleased with the matter introduced, as well as the dress of the journal, and I wish you all success in the effort to advance the interests of the profession. I noticed a favorable criticism in the 'Medical Record' of last week."

PROF. ROBERTS BARTHOLOW, M. D., of the Jefferson Medical College, Philadelphia, says: "I shall be pleased to send you a paper when I get through the work which I am compelled to do."

PROF. J. M. DA COSTA, M. D., of the same college and city: "I wish it (the PRACTITIONER) a most marked success."

PROF. THOS. H. CHANDLER, Dean of the Harvard University, Dental Department: "If you keep up the standard, your magazine will fill a void in our professional literature."

PROF. JAS. E. GARRETSON, M. D., D. D. S., Dean of the Philadelphia Dental College: "Let me wish you success in an enterprise which looks as if calculated to be of great use to the dentist. I have read each number of the journal with much satisfaction and have to apologize for my tardiness in acknowledging your kindness in sending it. I welcome the PRACTITIONER particularly from the standpoint of its medico-dental character. The more dentists learn of general medicine and surgery the better are they found qualified for work as specialists."

COL. J. J. WOODWARD, Surgeon U. S. Army: "I wish for your enterprise a useful and honorable career and all success."

PROF. WM. A. HAMMOND, M. D., N. Y., and formerly Surgeon-General of the U. S. Army: "I am pleased with the INDEPENDENT PRACTITIONER and wish you success." Later: "I will ere long send you a paper which may prove of some interest to your readers."

PROF. ISAAC J. WETHERBEE, D. D. S., President of the Boston Dental College: "A careful examination of the contents of the INDEPENDENT PRACTITIONER leads me to believe that it will be a very useful publication to the dental profession, and I hope that you may meet with large success."

SAM D. RAMBO, D. D. S., of Rio, Brazil: "I find the INDEPENDENT PRACTITIONER very good. The best journal out."

Space will allow of the mention of a few more names only of those who have sent us words of encouragement or promised us original papers: PROF. AUSTIN FLINT, Sr., M. D., PROF. FRANK H. HAMILTON, M. D., PROF. T. GAILLARD THOMAS, M. D., ALFRED L. LOOMIS, M. D., J. MARION SIMS, M. D., GEO. M. BEARD, and others in our city. PROF. WM. PEPPER, M. D., PROF. S. WEIR MITCHELL, M. D., RICHARD J. DUNGLISON, M. D., and PROF. CHAS. J. ESSIG, D. D. S., M. D., of Philadelphia.

THE

### Independent Practitioner

Is the only Monthly Journal published in New York City which treats of General Medicine.

It contains Original Papers and Clinics from the most eminent men in the Profession. Its Translations, Abstracts, Selections, Editorials, Current News, Book Notices, Progress of Medicine and Science throughout the world are not surpassed by any medical journal.

It is intended for the busy practitioner—general and special—who wants matter direct from the great medical centre and the metropolis of America.

Send $2.50 to us and try it for the present year. Address

COLUMBIA PUBLISHING CO.,
42 DUANE STREET, NEW YORK.

### Independent Practitioner:

A MONTHLY JOURNAL,

DEVOTED TO

MEDICINE, SURGERY, OBSTETRICS, DENTISTRY, PATHOLOGY AND POPULAR SCIENCE.

W. C. BARRETT, M. D., D. D. S.,

EDITOR.

**Contributors:**

| | | | |
|---|---|---|---|
| ALF. L. LOOMIS, M.D. | New York | J. J. CHISOLM, M.D. | Baltimore, Md. |
| FESSENDEN N. OTIS, M.D. | " | C. F. BEVAN, M.D. | " |
| F. H. BOSWORTH, M.D. | " | I. E. ATKINSON, M.D. | " |
| J. L. LITTLE, M.D. | " | H. MCGUIRE, M.D. | Richmond, Va. |
| J. WILLISTON WRIGHT, M.D. | " | F. P. PORCHER, M.D. | Charleston, S. C. |
| C. E. FRANCIS, D.D.S., M.D.S. | " | JAS. T. WHITTAKER, M.D. | Cincinnati, O. |
| A. J. C. SKENE, M.D. | Brooklyn, N. Y. | J. RANSOHOFF, M.D., F.R.C.S. | " |
| C. A. MARVIN, D.D.S. | " | F. FORCHHEIMER, M.D. | " |
| EDWIN T. DARBY, M.D., D.D.S. | Phila., Pa. | A. R. JACKSON, M.D. | Chicago, Ill. |
| P. S. WALES, M.D. | Surg.-Gen'l U. S. Navy | S. V. CLEVENGER, M.D. | " |
| H. F. CAMPBELL, M.D. | Augusta, Ga. | E. F. INGALS, M.D. | " |
| C. H. MASTIN, M.D., LL.D. | Mobile, Ala. | J. H. CARSTENS, M.D. | Detroit, Mich. |

COLUMBIA PUBLISHING COMPANY.

**$2.50 Per Annum in Advance. - - Single Copies, 25 Cents.**

Entered at the Post-Office, Buffalo, N. Y., as Second-Class matter.

### The Professional Man who does not read his Professional Literature, will Always Have Place in the Rear of his Profession.

---

### The Independent Practitioner—Vol. IV.

---

### Prospectus.

This Journal is published by an association of professional men who have no ulterior objects other than to afford to the profession an independent, outspoken Journal, which shall be the exponent of higher aims and a better practice.

Having no connection with any school, clique, or advertising firm, it will be impartial in its judgment of professional matters, and its appeals for support are directed to those who believe that a liberal profession should have an independent literature.

While not neglecting practical details, it will pay especial attention to those oral diseases that have usually been under the care of the general practitioner, but which more properly belong to the specialty of dentistry.

Its pages will be open to any member of the profession who has anything of interest to communicate, and it invites a free expression of views on all subjects medical or dental.

As each of the gentlemen forming the association is practically a member of the editorial corps, it will have abundant opportunity for collecting all the latest professional news and intelligence.

It will have a body of regular contributors, selected from the very best writers in the profession, and will thus be certain of an abundance of interesting original matter.

It will pay especial attention to reports of the meetings of dental societies, and its aim will be to publish an epitome of all important discussions of professional matters.

It will contain original abstracts and translations of all that is of moment in the foreign journals.

Its profits, if any, will be devoted to its improvement by adding needed illustrations, paying for valuable original communications, and enlarging its size whenever this becomes advisable.

To the accomplishment of the work thus outlined (relying upon the hearty approval of an appreciative profession), each of the publishers of the Independent Practitioner has pledged his best efforts.

Editorial communications should be addressed to Dr. W. C. Barrett, No. 11 West Chippewa St., Buffalo, N. Y. Send all business letters to Dr. William Carr, 35 West Forty-Sixth Street, New York.

| | | |
|---|---|---|
| ABBOTT, FRANK ...New York. | CARR, WILLIAM..New York. | JARVIE, WM. Jr. Brooklyn. |
| BARRETT, W. C..........Buffalo. | FRANCIS, C. E....New York. | NORTHROP, A. L.New York. |
| BODECKER, C. F. W..New York. | HILL, O. E..........Brooklyn. | PALMER, S. B......Syracuse. |

